# Homologous expression and purification of human HAX‐1 for structural studies

**DOI:** 10.1002/2211-5463.70131

**Published:** 2025-09-25

**Authors:** Mariana Grieben

**Affiliations:** ^1^ Institute of Biochemistry, Center of Structural and Cell Biology in Medicine University of Lübeck Germany

**Keywords:** Expi293F mammalian cells, HAX‐1, intrinsic disorder, posttranslational modifications, protein expression, protein purification

## Abstract

The human HAX‐1 protein is a ubiquitously expressed multifunctional protein that regulates various cellular processes through interactions with numerous cellular proteins and RNA. The purification protocol presented here is designed explicitly for HAX‐1 expressed in mammalian cells and yields high amounts of soluble HAX‐1 protein constructs N‐terminally fused to a cleavable superfolder GFP. This protocol will enable structural studies of HAX‐1, which is predicted to undergo posttranslational modifications and be partially disordered in the absence of a binding partner.

## Graphical abstract

This research protocol provides detailed instructions for cloning, expressing, and purifying large quantities of the intrinsically disordered human HAX‐1 protein, N‐terminally fused to a cleavable superfolder GFP, from mammalian cells. HAX‐1 is predicted to undergo posttranslational modifications and to interact with membranes, various cellular proteins, viral proteins, and RNA. This protocol aims to facilitate *in vitro* protein–protein interaction studies and structural analyses.

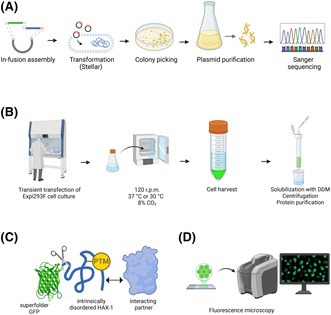

**Abbreviations**
CMC: critical micelle concentration
DDM: dodecyl‐β‐d‐maltoside
DTT: dithiothreitol
*E. coli*
: *Escherichia coli*
GFP: green fluorescent protein
HAX‐1: HS1‐associated protein X‐1
HEPES: 4‐(2‐hydroxyethyl)‐1‐piperazineethanesulfonic acid
HS‐1: hematopoietic cell‐specific protein 1
IMAC: immobilized metal affinity chromatography
IPTG: isopropyl β‐d‐1‐thiogalactopyranoside
ipTM: interface predicted template modeling
LB: Luria‐Bertani
MW: molecular weight
PEI: polyethylenimine
pLDDT: predicted local distance difference test
pTM: estimate of the global superposition metric template modeling score
SDS/PAGE: sodium dodecyl‐sulfate polyacrylamide gel electrophoresis
TEV: Tobacco etch virus
ε: molecular extinction coefficient

HS1‐associated protein X‐1 (Hax‐1) is a protein first discovered in 1997 as a binding partner of the hematopoietic cell‐specific protein 1 (HS‐1), a protein involved in the maturation of T cells [1]. Mutations within this gene cause autosomal recessive severe congenital neutropenia (Kostmann disease), a primary immunodeficiency syndrome associated with increased apoptosis in myeloid cells [2].

HAX‐1 is expressed ubiquitously and found in several cellular compartments such as mitochondria, the nuclear matrix, the endo‐ and sarcoplasmic reticulum, apical membranes, nuclear envelopes, lamellipodia, as well as associated with the actin cytoskeleton [1].

HAX‐1 is heavily spliced, leading to at least eight structurally distinct isoforms [3]. In addition, HAX‐1 has been reported to form homodimers (with antiapoptotic or pro‐apoptotic function depending on the isoform) and heterodimers, indicating a highly complex and manifold regulation of cell survival or death [4, 5]. Moreover, HAX‐1 is critical for maintaining the inner mitochondrial membrane potential and can rescue myeloid cells [2], cardiomyocytes [6], and neurons [7] from programmed cell death.

HAX‐1 has an N‐terminally localized putative EF‐hand‐like structure with calcium binding capacity [8], while the HAX‐1 C‐terminal tail is predicted to lack an ordered structure in the absence of a binding partner and is, therefore, expected to possess configurational adaptability. In numerous independent publications, the HAX‐1 C‐terminal tail has been described as associating with various cellular proteins, viral proteins, human RNA [9], and membranes [1, 10] in several cellular compartments. Moreover, Yin and co‐workers [11] proposed the first putative consensus binding motif, through multiple sequence alignment of interacting partners of HAX‐1, indicating specificity in binding.

Additionally, HAX‐1 has been reported to be involved in diverse cellular signaling pathways, including tumor cell survival, metastasis, and cell migration [12]. There is also some indication that several microRNAs may directly regulate the activity of HAX‐1 [13, 14, 15, 16, 17, 18].

Attempts to purify HAX‐1 produced in *E. coli* have led to the formation of highly aggregated proteins, commonly referred to as inclusion bodies, requiring refolding [4]. Moreover, theoretical predictions suggest that the HAX‐1 monomer is primarily an intrinsically disordered protein that undergoes posttranslational modifications.

Based on the current understanding of HAX‐1 and the predicted posttranslational modifications of HAX‐1 in eukaryotic cells, a protocol was established in this study to produce large quantities of HAX‐1 isoform 1 constructs in mammalian cells. This protocol aims to facilitate structural studies of HAX‐1 isoform 1 in complex with a binding partner, through single‐particle cryo‐electron microscopy or crystallography. It also supports intracellular localization and co‐localization studies through fluorescence microscopy.

## Materials

### Cloning and plasmid purification

1 Synthetic homo sapiens HAX1 gene purchased from Twist Bioscience (UniProtKB O00165).
1 Superfolder GFP [19].
2 pcDNA3.1 vector (Thermo Fisher Scientific, Waltham, MA, USA #V79020).
3 Platinum™ SuperFi Green PCR Mastermix (Thermo Fisher #12369010).
4 In‐Fusion Snap Assembly cloning kit and Stellar chemically competent cells (Takara Bio, Kusatsu, Shiga, Japan #638945).
5 Thermocycler.
6 Agarose gel electrophoresis.
7 Gel and PCR Clean‐up kit (Macherey‐Nagel, Düren, Germany #740609).
8 42 °C water bath.
9 Sterile 1.5 and 15 mL centrifuge tubes.
10 LB (Luria‐Bertani) liquid medium: 10 g tryptone, 5 g NaCl, 5 g yeast extract in 1 L ddH_2_O, and pH 7.2–7.5 (autoclave for 15 min at 120 °C).
11 LB agar (Luria–Bertani broth with 1.5% agar–agar) plates supplemented with antibiotics.
12 Antibiotic: ampicillin (AppliChem, Darmstadt, Germany #A0839).
13 Glass Erlenmeyer flasks.
14 Laboratory shaker with thermoregulation.
15 Refrigerated centrifuge and centrifuge tubes.
16 NucleoSpin Plasmid kit (Macherey‐Nagel #740588).
17 NucleoBond Xtra Midi EF kit (Macherey‐Nagel #740420).
18 Spectrophotometer (DeNovix DS‐11).

### Protein expression

1. Laminar flow cabinet.
2. One vial of cryopreserved Expi293F mammalian cells (Thermo Fisher #A14527).
3. FreeStyle™ 293 Expression Medium (Thermo Fisher #12338018).
4. 37 °C water bath.
5. Corning^®^ Erlenmeyer cell culture flasks (Merck KGaA, Darmstadt, Germany #CLS431147).
6. Sterile serological pipettes.
7. Sterile and filtered pipette tips.
8. Sterile 1.5, 2, 15, and 50 mL centrifuge tubes.
9. ExpiFectamine 293 transfection kit (Thermo Fisher #A14525).
10. PEI STAR™ transfection reagent (Tocris, Bristol, UK #7854).
11. Sodium butyrate (Carl Roth, Karlsruhe, Germany #1441.1).
12. CO_2_ orbital shaker with thermoregulation.
13. Automated cell counter (Countess II, Invitrogen).
14. Refrigerated centrifuge and centrifuge tubes.
15. Liquid nitrogen.

### Protein purification

1. Dounce homogenizer.
2. Syringes and PES Membrane Filters (SARSTEDT, Nümbrecht, Germany #83.1826.001) for sterile filtration of buffers.
3. Sterile 1.5, 2, 15, and 50 mL centrifuge tubes.
4. Tobacco etch virus (TEV) protease [20].
5. Buffer A: 50 mm HEPES pH 7.5, 500 mm NaCl, 5 mm CaCl_2_, 5% glycerol, Roche cOmplete EDTA‐free protease inhibitor cocktail.
6. n‐dodecyl‐ß‐D‐maltoside (DDM) (Carl Roth #CN26.1).
7. Buffer B: 50 mm HEPES pH 7.5, 500 mm NaCl, 5 mm CaCl_2_, 5% glycerol, 3 × critical micelle concentration (CMC) DDM, Roche cOmplete EDTA‐free protease inhibitor cocktail.
8. Buffer C: 50 mm HEPES pH 7.5, 500 mm NaCl, 5 mm CaCl_2_, 5% glycerol, and 3 × CMC DDM.
9. Buffer D: 50 mm HEPES pH 7.5, 500 mm NaCl, 5 mm CaCl_2_, 5% glycerol, 3 × CMC DDM, and 500 mm Imidazole.
10. Refrigerated centrifuge and centrifuge tubes.
11. Empty chromatography column.
12. TALON^®^ metal affinity resin (Takara Bio #635502).
13. PD MidiTrap™ G‐25 columns (Merck KGaA #28918008).
14. Spectrophotometer (DeNovix DS‐11).
15. SDS/PAGE apparatus, 10% or 15% SDS/PAGE gels, 5× SDS/PAGE sample loading buffer containing dithiothreitol (DTT), and Coomassie Brilliant Blue staining solution.
16. PageRuler™ Plus Prestained Protein Ladder (Thermo Fisher Scientific #26619).
17. Liquid nitrogen.

## Methods

### Cloning of HAX‐1

**Fig. 1:**
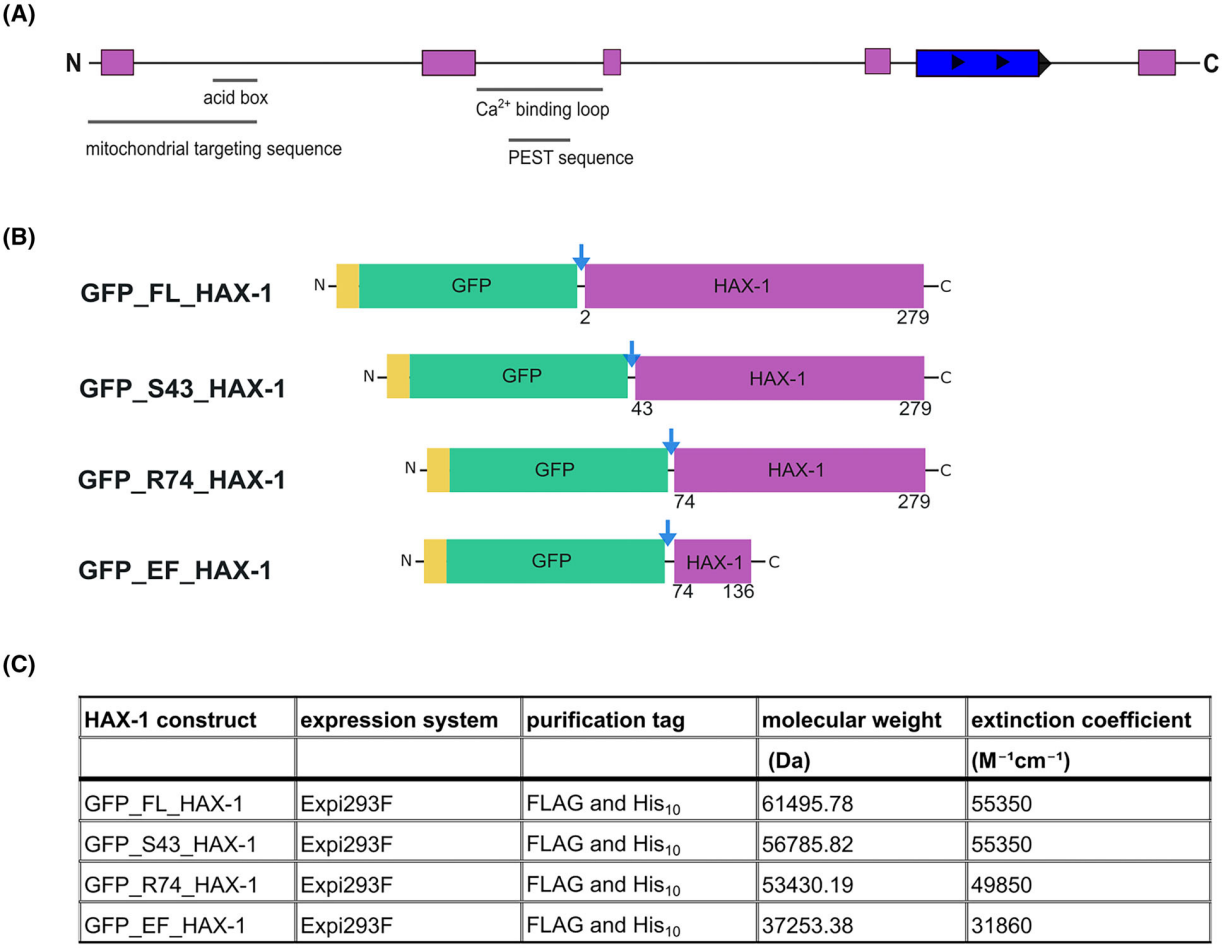
Construct design of human HAX‐1 for expression in mammalian cells. (A) Domain organization of human HAX‐1 isomer 1. Predicted α‐helices are shown as purple boxes and predicted β‐sheets as blue boxes. The locations of the mitochondrial targeting sequence [21], the acid box [22], the calcium‐binding region [8, 23], and the PEST sequence [24] are shown. (B) Designed and cloned constructs of HAX‐1 (Ser 1 to Arg 279, Ser 43 to Arg 279, Arg 74 to Arg 279, and Arg 74 to His 136) are shown as purple boxes. Purification tags are shown as yellow boxes. Superfolder GFP is shown as a green box. The TEV protease cleavage sites are marked with blue arrows. (C) Table summarizing the cloned constructs.

The Homo sapiens HAX1 gene was cloned into a pcDNA3.1 vector using the Platinum™ SuperFi Green PCR Mastermix and In‐Fusion Snap Assembly, per the manufacturer's instructions (Tips & Tricks 2). Primers were designed with the In‐Fusion Cloning tool within SnapGene (https://www.snapgene.com/series/simulate‐in‐fusion‐cloning). In the first construct, termed GFP_FL_HAX‐1, the start codon is followed by a FLAG purification tag, a His_10_‐purification tag, the DNA sequence of superfolder GFP, a Tobacco Etch Virus (TEV) cleavage site, and the full‐length sequence of human HAX‐1 isoform 1. Additionally, two N‐terminally truncated HAX‐1 constructs were cloned, starting at serine 43 (GFP_S43_HAX‐1) or arginine 74 (GFP_R74_HAX‐1). The fourth construct contained only the putative EF‐hand‐like domain (arginine 74 to histidine 136) and was designated GFP_EF_HAX‐1 (Fig. 1). All constructs were transformed into Stellar chemically competent cells, and the transformants were grown on LB agar plates supplemented with 100 μg/mL ampicillin (Tips & Tricks 3).

### Plasmid amplification and purification for Sanger sequencing

1. Pick a single colony of Stellar chemically competent cells transformed with plasmids of GFP_FL_HAX‐1, GFP_S43_HAX‐1, GFP_R74_HAX‐1, and GFP_EF_HAX‐1 from LB agar plates.
2. Inoculate 5 mL of LB liquid medium with a single colony and grow overnight at 37 °C and 180 r.p.m. in the presence of 100 μg/mL ampicillin.
3. Perform plasmid purification using the NucleoSpin Plasmid kit.
4. Confirm the correctness of all constructs through Sanger sequencing.

### Plasmid amplification and purification for transient expression in Expi293F

1. Transform positive clones into Stellar chemically competent cells, and grow on LB agar plates supplemented with 100 μg/mL ampicillin.
2. Inoculate 100 mL of LB liquid medium with a single colony and grow overnight at 37 °C and 180 r.p.m. in the presence of 100 μg/mL ampicillin.
3. Perform plasmid purification using the NucleoBond Xtra Midi EF kit.

### Small‐scale expression screening of HAX‐1 in Expi293F cells

1. Transiently transfect 10 mL of Expi293F cell culture (2 × 10^6^ cells·mL^−1^) with one of the HAX1 plasmids using the ExpiFectamine™ 293 transfection kit (Tips & Tricks 5–12).
2. Culture transfected cells in an orbital shaker at 37 °C or 30 °C and 120 r.p.m. with 8% CO_2_.
3. Harvest cells (3 mL of the culture) 48 h, 72 h, or 5 days post‐transfection by centrifugation at 900 g for 20 min at 4 °C.
4. Flash‐freeze pellets in liquid nitrogen.
5. Store pellets at −80 °C.

Note: Expi293F™ cells were chosen due to their high transfectability, resulting in significantly greater protein yields compared to standard 293 cell lines. Furthermore, these cells are engineered for optimal growth in suspension culture, allowing them to reach high cell densities. However, protein expression can also be carried out in various other mammalian cell lines.

### Small‐scale purification of HAX‐1 constructs

1. Resuspend each 3 mL pellet in 0.9 mL buffer A and lyse the cells using a Dounce homogenizer.
2. Add DDM to a final concentration of 1% (w/v) and rotate at 4 °C for 1 h.
3. Centrifuge at 18 000 ***g*** and 4 °C for 60 min.
4. Add 50 μL TALON® metal affinity resin to the supernatant and rotate the tube at 4 °C to capture the His‐tagged protein.
5. Transfer to an empty chromatography column and wash with 1600 μL of buffer C supplemented with 30 mm imidazole.
6. Elute with buffer D.
7. Analyze samples by sodium dodecyl sulfate polyacrylamide gel electrophoresis (SDS/PAGE).

**Fig. 2:**
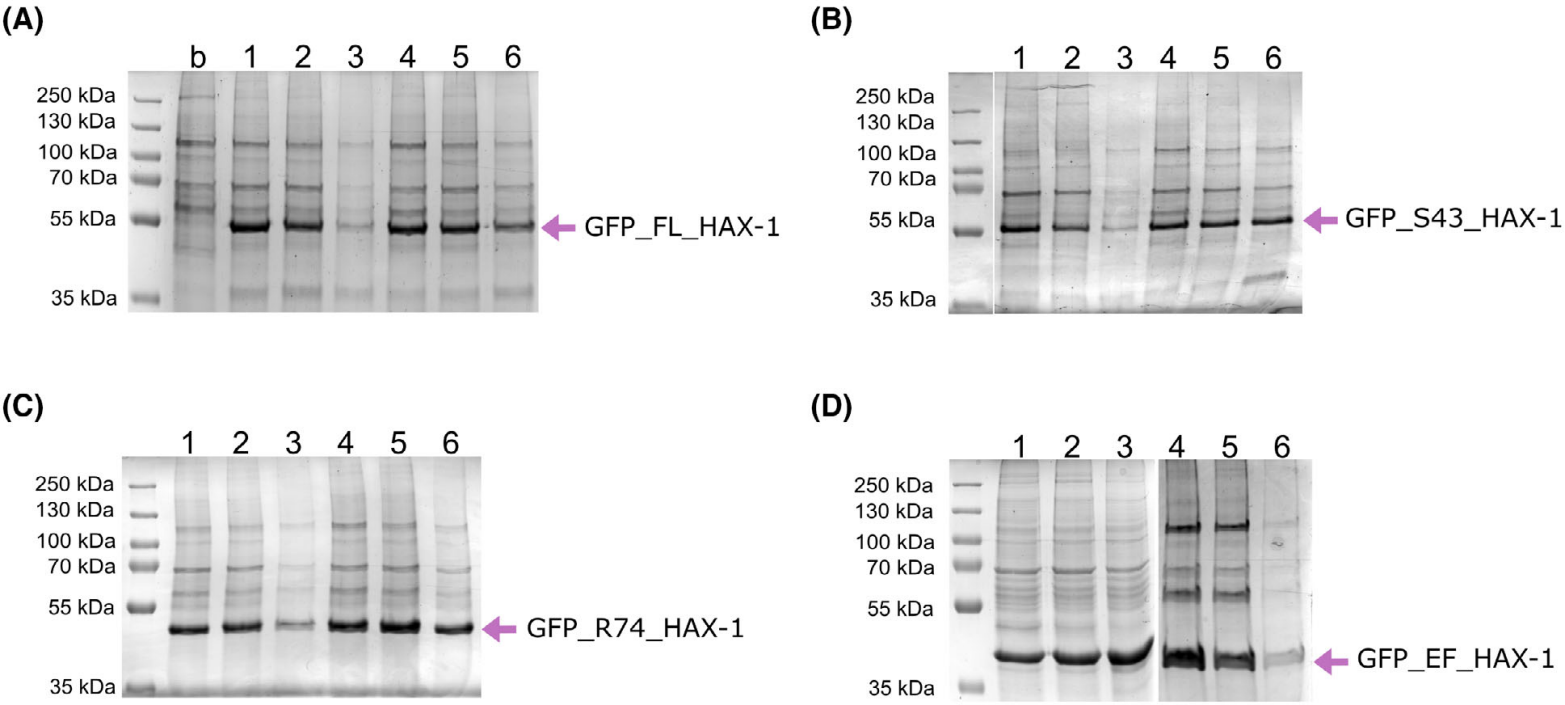
Small‐scale expression and purification of human HAX‐1 from mammalian cells. (A) GFP_FL_HAX‐1. 1: 48 h expression at 30 °C; 2: 72 h expression at 30 °C; 3: 120 h expression at 30 °C; 4: 48 h expression at 37 °C; 5: 72 h expression at 37 °C; 6: 120 h expression at 37 °C. (B) GFP_S43_HAX‐1 (grouped from different parts of the same gel). (C) GFP_R74_HAX‐1. (D) GFP_EF_HAX‐1 (grouped from two gels).

Since HAX‐1 has been proposed to associate with membranes and other cellular proteins, including integral membrane proteins, small‐scale purifications were carried out in the presence of the detergent DDM to solubilize membranes. DDM is a mild, non‐ionic, and non‐denaturing detergent commonly used for solubilizing membrane proteins. High levels of HAX‐1 expression were observed for all screened constructs at both 30 °C and 37 °C, with the optimal expression time ranging from 48 to 72 h (Fig. 2).

### Large‐scale expression of HAX‐1

1. Transfect 100 mL of Expi293F cell culture (2 × 10^6^ cells·mL^−1^) with 100 μg pDNA and 300 μL PEI STAR™ at 1 mg·mL^−1^, following the manufacturer's instructions (Tips & Tricks 6).
2. Grow cells in an orbital shaker for 24 h at 37 °C and 120 r.p.m. with 8% CO_2_.
3. Add sodium butyrate to a final concentration of 5 mm.
4. Harvest cells 48 h later by centrifugation at 900 ***g*** for 20 min at 4 °C.
5. Flash‐freeze in liquid nitrogen.
6. Store at −80 °C.

### Fluorescence microscopy

**Fig. 3:**
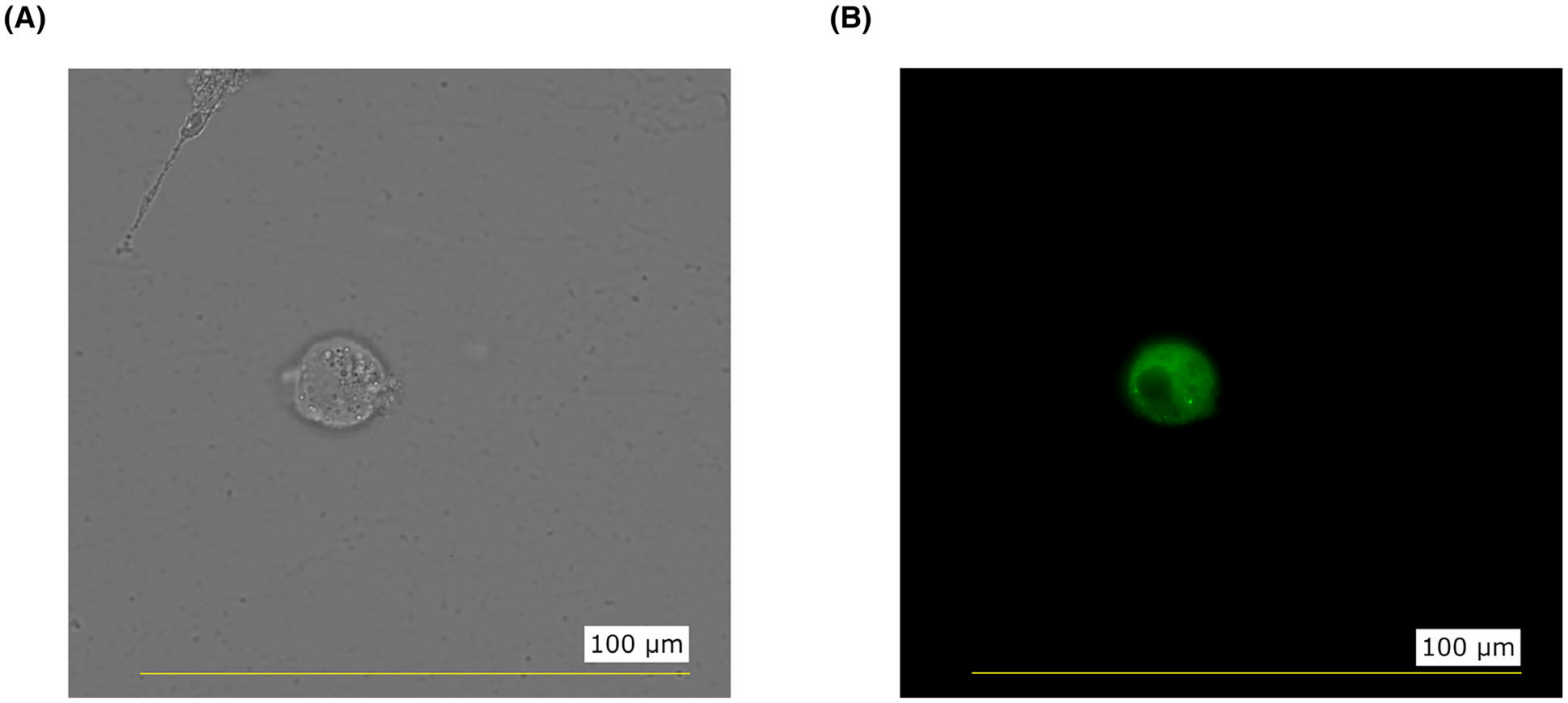
Fluorescence imaging. (A) Single Expi293F cell transiently transfected with the GFP_FL_HAX‐1 construct. (B) The fluorescence of the GFP in the cell is shown (*n* > 3 cells). Scale bar: 100 μm.

A few cells from a suspension culture were placed on a microscope glass slide and covered with a glass coverslip. Fluorescence was observed using a fluorescence microscope (Keyence, BZ‐X) and a 60× objective (Fig. 3).

### Large‐scale purification of HAX‐1

**Fig. 4:**
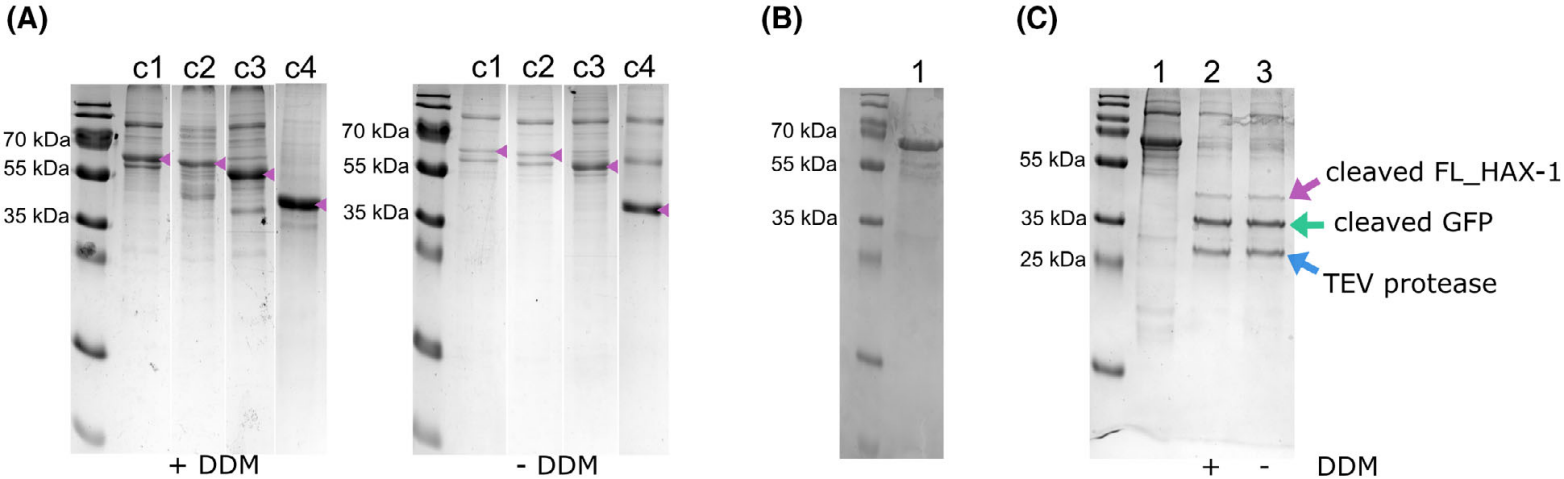
Large‐scale purification of human HAX‐1 from mammalian cells. (A) Purification of the constructs c1 (GFP_FL_HAX‐1), c2 (GFP_S43_HAX‐1), c3 (GFP_R74_HAX‐1), and c4 (GFP_EF_HAX‐1) in the presence of DDM (left) and in the absence (right) (grouped from different gels). (B) Large‐scale purification of GFP_FL_HAX‐1 (lane 1) using the FLAG‐tag. (C) Large‐scale purification of GFP_FL_HAX‐1 (lane 1) using the His‐tag, followed by treatment with TEV protease in the presence of DDM (lane 2) and the absence (lane 3). Relevant bands are highlighted.

Purification can be performed using either ANTI‐FLAG M2 Affinity Gel (Fig. 4B and Tips & Tricks 6) or TALON metal affinity resin (Fig. 4C). The following steps describe the purification process using TALON.

1. Resuspend one frozen pellet in 5 mL buffer A (Tips and Tricks 4).
2. Lyse the cells using a Dounce homogenizer.
3. Add DDM to a final concentration of 1% (w/v) and rotate in the cold room for 1 h.
4. Increase the volume to 20 mL with buffer B.
5. Centrifuge at 45 000 ***g*** and 4 °C for 60 min.
6. Add TALON^®^ metal affinity resin to the supernatant and rotate the tube at 4 °C to capture the His‐tagged protein.
7. Transfer the mixture to an empty chromatography column and wash with 30 CV of buffer C supplemented with 30 mm imidazole.
8. Elute with buffer D.
9. Buffer exchange into buffer C with a PD MidiTrap™ G‐25 column.
10. Determine the protein concentration with a spectrophotometer, according to the user guide (https://www.denovix.com/ds‐11‐series‐user‐guide/). MW and ε can be calculated with the ProtParam online tool (https://web.expasy.org/protparam/) [25].
11. Analyze by SDS/PAGE.

To study the impact of DDM on the final yield, large‐scale purifications were conducted in the presence and absence of the detergent DDM, affecting the constructs differently. Purifications of GFP_HAX‐1, GFP_S43_HAX‐1, and GFP_R74_HAX‐1 conducted without DDM yielded significantly less fusion protein compared to those carried out with the detergent, whereas GFP_EF_HAX‐1 remained unaffected (Fig. 4A). This observation is best explained by the fact that the GFP_EF_HAX‐1 construct lacks the C‐terminal tail predicted to interact with membranes and other cellular proteins.

Cleavage of the tagged GFP from the full‐length HAX‐1 protein produces three distinct bands on the SDS/PAGE corresponding to the HAX‐1 protein carrying an additional serine residue on the N terminus (31.58 kDa), the tagged GFP (29.94 kDa), and the TEV protease (28.62 kDa). Cleavage was performed in the presence and absence of DDM, yielding the same result (Fig. 4C).

In summary, solubilizing the cellular membranes with DDM appears to enhance the yield of extracted HAX‐1; however, DDM is not necessary for any of the subsequent purification steps. On average, up to 1.2 mg of GFP_HAX‐1 can be obtained from 100 mL of cell culture. This protocol can be scaled up as needed to produce the required amount of protein.

### Sodium dodecyl‐sulfate polyacrylamide gel electrophoresis

1. Mix samples with 5× SDS loading buffer containing DTT.
2. Heat for 10 min at 98 °C.
3. Run 10% or 15% SDS/PAGE.
4. Stain gel with Coomassie Brilliant Blue Solution (Figs 2 and 4).

### HAX‐1 3D‐structure prediction

**Fig. 5:**
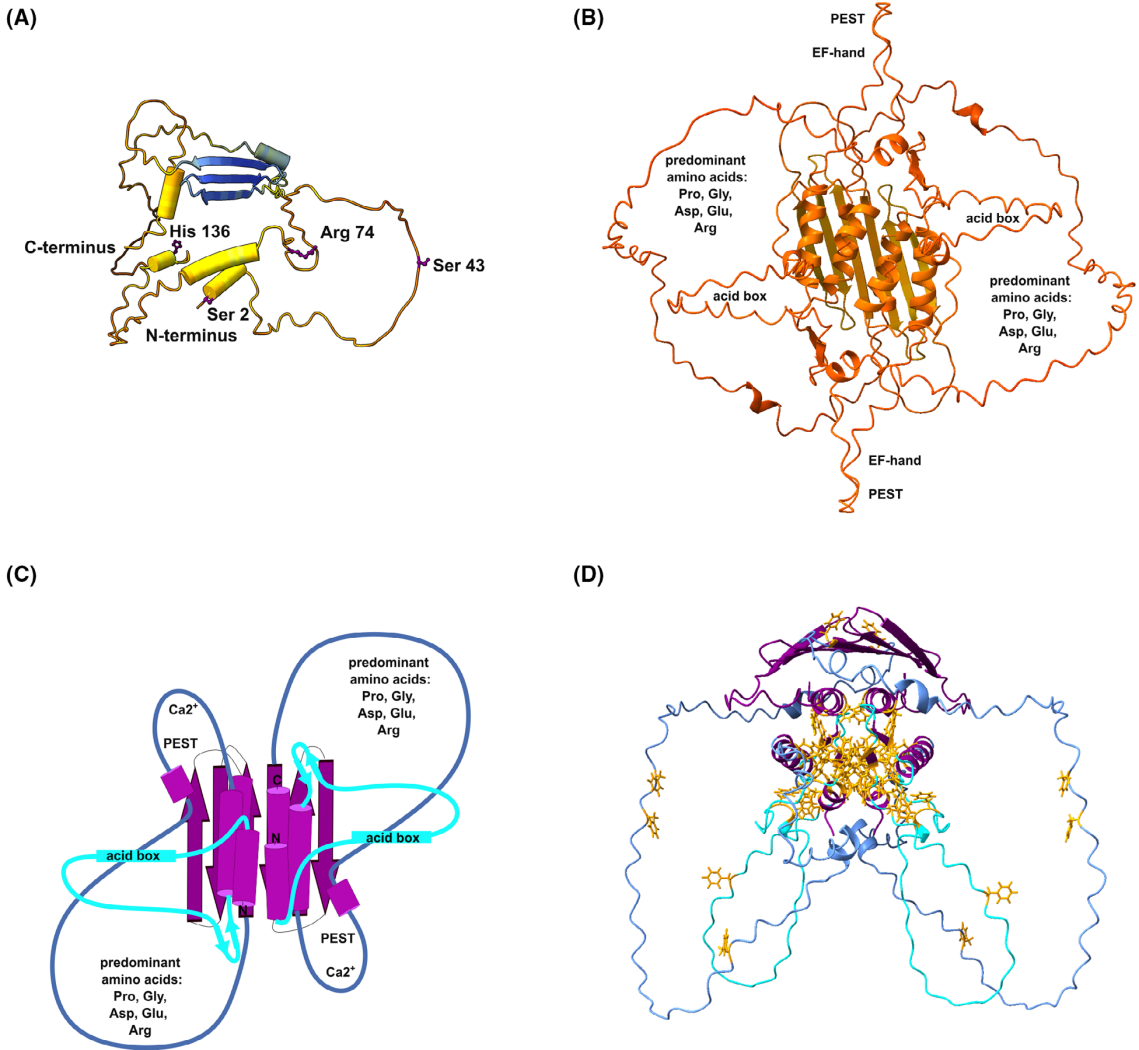
Human HAX‐1 isoform 1 models predicted by AlphaFold. (A) Model of human HAX‐1 isomer 1 monomer predicted by AlphaFold within https://www.tamarind.bio/. The model is shown with high‐confidence residues colored blue and lower‐confidence residues in yellow, orange, and red. Relevant residues (Ser 2, Ser 43, Arg 74, and His 136) for construct design are highlighted. (B) AlphaFold model of the human HAX‐1 isomer 1 dimer colored again according to per‐residue confidence. (C) Cartoon representation of human HAX‐1 isomer 1 dimer. The predicted α‐helices and β‐sheets are colored purple. Light and dark blue lines present segments predicted to be disordered. (D) Phenylalanine residues are shown in yellow.

AlphaFold v2.3.2 (implemented by ColabFold) [26, 27] within Tamarind Bio (https://www.tamarind.bio/) [28] was used to predict the three‐dimensional structure of the full‐length human HAX‐1 isoforms 1 monomer and dimer (Fig. 5). The sequence was taken from UniProt [29]. Structure prediction was performed with the default settings, but the number of cycles was increased to 20, generating five models each. All models were analyzed.

### Amino acid composition of HAX‐1

**Fig. 6:**
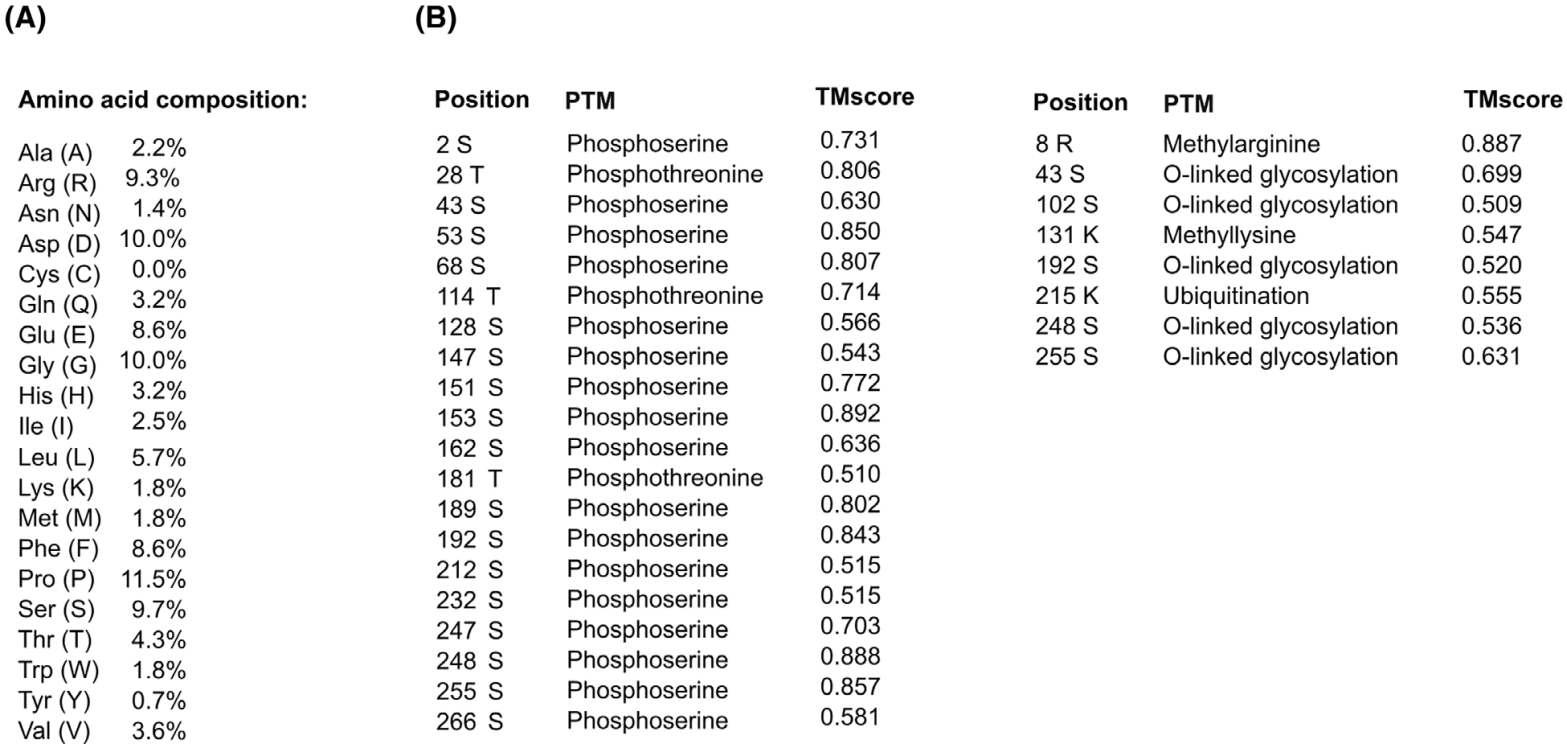
Amino acid composition and predicted posttranslational modifications of human HAX‐1. (A) Amino acid composition of human HAX‐1 isomer 1 and (B) posttranslational modifications predicted by MusiteDeep [31].

The amino acid composition of HAX‐1 isomer 1 was determined using the ProtParam [25] program within Expasy [30] (Fig. 6A).

### Prediction of posttranslational modifications

Posttranslational modifications within the HAX‐1 protein isoform 1 were predicted with MusiteDeep [31] (Fig. 6B).

## Discussion

In this study, a protocol for producing high yields of the intrinsically disordered full‐length human HAX‐1 isoform 1 from mammalian cells, N‐terminally fused to a superfolder GFP, was established, thereby allowing posttranslational modifications to occur during protein expression. The superfolder GFP at the N terminus of HAX‐1 improves its solubility, while the HAX‐1 C‐terminal tail, predicted to interact with other proteins and membranes, remains free.

HAX‐1 is predicted to be predominantly disordered without a binding partner. It contains an N‐terminal EF‐hand‐like structure with calcium binding capacity [8] and a C‐terminal tail that associates with protein and RNA partners [9]. Increased local calcium levels likely cause conformational changes in HAX‐1, which may affect its interactions with binding partners. Moreover, the anticipated posttranslational modifications at different sites, such as phosphorylation, are likely to either enhance or inhibit the binding of a partner, thus regulating protein function.

With the aid of fluorescence microscopy, HAX‐1 has been observed in the cytosol [32] and P‐bodies [33]. Additionally, it can associate with membranes via its C terminus [10]. To effectively purify the homologously produced HAX‐1, it is essential to use a mild, non‐ionic, non‐denaturing detergent that solubilizes all cellular membranes. This method maximizes the extraction of HAX‐1 from the cells, as shown by the results of this study.

It is unclear whether HAX‐1 functions in the cell exclusively as a dimer, both as a dimer and a monomer, or solely as part of a larger multi‐protein complex. Isolated HAX‐1 is predicted to be predominantly intrinsically disordered. Supporting this prediction, earlier experiments in which HAX‐1 was purified from *E. coli* showed the formation of inclusion bodies, necessitating refolding. Interestingly, these purified isomers of HAX‐1 have been reported to form both homo‐ and heterodimers upon refolding [4], but the dimerization interface has not yet been identified. Therefore, AlphaFold was utilized to predict the three‐dimensional structure of the full‐length human HAX‐1 monomer and dimer.

The three‐dimensional structure of the full‐length human HAX‐1 monomer, as predicted by AlphaFold, indicates that the protein is predominantly structurally disordered. However, AlphaFold predicts a three‐stranded β‐sheet and a few short α‐helices with pLDDT values exceeding 50. On the other hand, pLDDT values below 50 are generally a strong predictor of disorder, suggesting that such a region is either unstructured in physiological conditions or only structured as part of a complex. In the case of HAX‐1, the structural disorder of the remaining segments is best explained by the amino acid composition of isomer 1. The most abundant amino acid in HAX‐1 is proline (11.5%), followed by glycine and aspartate (each 10%), serine (9.7%), arginine (9.3%), phenylalanine (8.6%), and glutamate (8.6%). Proline and glycine are known as helix breakers. Aspartate and glutamate are negatively charged, while arginine is positively charged at physiological pH. The negatively charged amino acids (18.6%) are approximately 1.8 times more abundant than the positively charged amino acids (11.1%). Moreover, several residues are predicted to undergo posttranslational modifications such as phosphorylation and glycosylation, possibly regulating protein function. Overall, the disordered regions within HAX‐1 are enriched in proline residues, charged amino acids, and serine residues.

Striking, however, is the number of phenylalanine residues and the fact that these agglomerate within the secondary structure predicted by AlphaFold. Phenylalanine is classified as a hydrophobic amino acid. The predicted phenylalanine‐rich helices are too short to span the membrane, but they might encourage self‐association in an aqueous environment, leading to homo‐ or heterodimerization, or they could engage in membrane association without actually spanning it. Notably, phenylalanine‐rich helices are also known to facilitate folding in parvalbumin, a soluble calcium‐binding protein that shares some similarities with HAX‐1 [8]. However, there is no indication that the phenylalanine‐rich helices facilitate the folding of the HAX‐1 monomer.

In the dimeric HAX‐1 model created by AlphaFold, on the other hand, the secondary structures self‐associate due to the hydrophobic nature of the phenylalanine residues, forming a structured core from which disordered loops extend. However, the confidence scores for this model are low (pLDDT <60, pTM <0.5, and ipTM <0.3). Several factors can explain this. First, most of the protein remains intrinsically disordered in the dimeric model, creating a low pLDDT score, which negatively impacts the ipTM score. Second, potential posttranslational modifications or the binding of calcium ions were not taken into consideration. Third, the model was created in the absence of a binding partner. Fourth, all amphipathic helices were used to model a structured core, although not all helices may actually participate. In fact, the final C‐terminal tail of HAX‐1 has been proposed to associate with lipid bilayers, while a significant portion of HAX‐1 remains dynamic and does not interact with lipid membranes [10]. How the C terminus of HAX‐1 interacts with membranes remains to be clarified.

The GFP, used in this study, serves four main purposes. First, it enhances the solubility of the intrinsically disordered HAX‐1 during expression. Secondly, the enhanced solubility of monomeric HAX‐1 could facilitate the binding to a partner *in vitro*. The GFP could be removed at this point. Third, it adds a green color to the colorless HAX‐1 protein, which allows direct monitoring of solubility and aggregation. Fourth, the protein construct can be directly employed in experiments to determine the localization of HAX‐1 in the cell and the co‐localization with selected partners, using fluorescence microscopy.

Although the production of the full‐length HAX‐1 was the main purpose of this study, three truncated constructs were also generated to decrease the amount of intrinsic disorder. These constructs expressed and purified well; however, *they* did not reveal any clear advantage compared to the full‐length protein. These constructs could, however, be used in experiments where these regions are to be studied in more detail.

In conclusion, the human HAX‐1 protein is predicted to undergo posttranslational modifications, likely impacting the protein's function and ability to interact with membranes, RNA, and cellular proteins. Therefore, a purification protocol for several HAX‐1 isoform 1 constructs expressed in mammalian cells was established, resulting in very high yields of cleavable fusion protein. This protocol will support structural studies of HAX‐1 in complex with a binding partner, using either single‐particle cryo‐electron microscopy or crystallography, as well as intracellular (co‐)localization studies via fluorescence microscopy. Based on our findings, the next steps will involve mass spectrometry analysis of posttranslational modifications and screening to identify optimal buffer conditions that promote folding, dimerization, and protein complex formation with an interacting partner. Determining a HAX‐1 structure likely requires a binding partner rather than relying on advanced model predictions.

## Tips and Tricks

1. Chemicals for which no specific supplier was specified can be purchased from any vendor. All chemicals should be of cell culture grade or high purity.
2. The user guides for the Platinum™ SuperFi Green PCR Mastermix and the In‐Fusion Snap Assembly cloning kit are available here: https://www.thermofisher.com/order/catalog/product/de/en/12369010 and here https://www.takarabio.com/learning‐centers/cloning/in‐fusion‐cloning‐general‐information/in‐fusion‐cloning‐overview.
3. All tubes and flasks for culturing *E. coli* cells must be sterile.
4. All centrifuges and buffers used for protein purification should be pre‐cooled to 4 °C, and the purification should be performed at 4 °C.
5. The user guide that comes with the Expi293F cell vial is available here: https://www.thermofisher.com/order/catalog/product/de/en/A14527.
6. The user guides for the PEI STAR™ transfection reagent are available here: https://www.tocris.com/products/pei‐transfection‐reagent_7854.
7. Researchers must follow laboratory rules and safety guidelines when handling Expi293F cells (https://www.thermofisher.com/de/en/home/references/gibco‐cell‐culture‐basics.html).
8. Expi293F cells in suspension cultures should be maintained between 0.2 × 10^6^ cells·mL^−1^ and 2 × 10^6^ cells·mL^−1^ in FreeStyle™ 293 Expression Medium at 37 °C and 8% CO_2_ on a shake platform set to 120 r.p.m.
9. All flasks for culturing Expi293F cells must be sterile and endotoxin‐free.
10. The Expi293F cell culture volume should not exceed 20% of the flask's total volume to allow for aeration and gas exchange.
11. Cell viability at the time of transient transfection should be greater than 95%.
12. Transient transfections must be carried out under sterile conditions and require the use of a laminar flow hood.
13. The technical Bulletin for the FLAG^®^ M2 Affinity Gel is available here: https://www.sigmaaldrich.com/DE/en/product/sigma/a2220.

## Conflict of interest

The author declares that she has no known competing financial interests or personal relationships that could have appeared to influence the work reported in this paper.

## Author contributions

MG designed research, conducted research, analyzed data, and wrote the paper.

## Data Availability

Data will be made available upon request.
